# Clinical characteristics and outcomes of COVID-19 infection in nine pregnant women: a report from a sub-Saharan African country, Senegal

**DOI:** 10.11604/pamj.supp.2020.35.2.23736

**Published:** 2020-06-02

**Authors:** Abdoul Aziz Diouf, Khardiata Diallo Mbaye, Mamour Gueye, Daouda Thioub, Ndama Niang, Christelle Yonta Dekou, Mame Diarra Ndiaye Gueye, Moussa Diallo, Magatte Mbaye, Marie Edouard Faye Dieme, Alassane Diouf, Moussa Seydi

**Affiliations:** 1 Department of Gynecology and Obstetrics, Pikine National Hospital Center, Dakar, Senegal; 2 Infectious and Tropical Diseases Department, Fann University Hospital, Dakar, Senegal; 3 Department of Gynecology and Obstetrics, Philippe Maguilen Senghor Health Center, Dakar, Senegal; 4 Department of Gynecology and Obstetrics, Tambacounda Regional Hospital, Tambacounda, Senegal; 5 Gynecological and Obstetric Clinic, Aristide Le Dantec Hospital, Dakar, Senegal

**Keywords:** COVID-19, SARS-COV-2, coronavirus, pregnancy, Senegal

## Abstract

**Introduction:**

To investigate the clinical characteristics of COVID-19 in pregnancy in Senegal.

**Methods:**

This was a cross-sectional and descriptive study of all cases of COVID-19 including nine pregnant women who were admitted in COVID-19 treatment centers in Senegal from March 2 to May 15, 2020. SARS-COV-2 infection was confirmed by PCR. Patients’ characteristics, clinical features, treatment and outcome were obtained with a customized data collection form.

**Results:**

The frequency of the association COVID-19 and pregnancy was 0.5%. The age range of the patients was 18-42 years with an average 28 years, and the range of gestational weeks at admission was 7 weeks to 32 weeks. None of the patients had underlying diseases. All the patients presented with a headache and only four of them had fever. Other symptoms were also observed: two patients had a cough, two had rhinorrhea, and two patients reported poor appetite. The median time to recovery was 13.6 days, corresponding to the number of days in hospital. None of the nine pregnant women developed severe COVID-19 pneumonia or died.

**Conclusion:**

Pregnant women appear to have the same contamination predispositions and clinical features of SARS-COV-2 infection as the general population. This study shows no evidence that pregnant women are more susceptible to infection with coronavirus.

## Introduction

On January 30, 2020, at the end of the second meeting of the Emergency Committee for International Health Regulations (IHR), the WHO qualified COVID-19 “a public health emergency of international concern”. On March 11, 2020, the Director General of WHO described it as a “Pandemic” during his speech [1]. On March 2, 2020, Senegal notified its first confirmed case of COVID-19. Since then, the number of cases has continued to grow with the regular occurrence of new community cases. Even though pregnant women are not among the risk groups linked to the COVID-19 pandemic, they can still be infected with harmful consequences on pregnancy. After 3 months of presence in Senegal, it becomes important to investigate the association COVID-19 and pregnancy in Senegal. The objective of this paper is to investigate the clinical characteristics of COVID-19 in pregnancy.

## Methods

This is a cross-sectional and descriptive study of COVID-19 pneumonia including nine pregnant women who were admitted in COVID-19 treatment centers in Senegal from March 2 to May 15 2020. COVID-19 was confirmed by laboratory findings using PCR (with maternal throat swab samples that were positive for SARS-CoV-2) from the Department of Clinical Laboratory of Pasteur Institute, Dakar, Senegal. We retrieved clinical records and laboratory findings for 9 pregnant women. Patients´ characteristics, clinical features, treatment and outcome were obtained with a customized data collection form. Statistical analysis was carried out with Microsoft Excel 2019. Continuous variables were expressed using their position parameters while qualitative variables were expressed in percentages.

## Results

As of May 15, 2020, 2,105 people were positive for COVID-19 in Senegal including 9 pregnant women leading to a rate of 0.5%. The age range of the patients was 18-42 years with an average of 28 years. The range of gestational weeks at admission was 7 weeks to 32 weeks. Two women were in their first trimester, five of them were in their second trimester, while two patients were in their third trimester. None of the patients had underlying diseases such as diabetes, chronic hypertension, asthma, HIV/AIDS or cardiovascular disease. All the patients presented with a headache and only four of them had fever without chills. Other symptoms were also observed: two patients had a cough, two had rhinorrhea, and two patients reported poor appetite. The diagnosis was confirmed by PCR for all pregnant women. Three out of nine patients were administered hydroxychloroquine and azithromycin. The median time to recovery was 13.6 days. Patients´ characteristics, treatment and outcomes are displayed in Table 1. The pregnancy outcome was fortunate for 8 out of the nine pregnant women. The youngest 18-year-old patient had experienced bleeding from the start of her hospitalization. Complete miscarriage was then diagnosed. Currently, all of the patients in our series benefit from regular pregnancy follow-up as part of the refocused antenatal care program. None of the nine pregnant women developed severe COVID-19 pneumonia or died, as of May 15th, 2020.

**Table 1:** Clinical and laboratory characteristics, treatments and perinatal outcomes in 9 patients with Coronavirus Disease-19 in Senegal

Clinical features											Treatment		Outcome
#	Age	Parity	GA	Origin	Comorbidity	Fever	Headache	Rhinorrhea	Diarrhea	Cough	HC	AZ	Time to recovery
**N°1**	23	1	25	Community	None	-	+						8
**N°2**	42	6	14	Spouse	None	+	+						21
**N°3**	25	1	20	Spouse	None	-	+	+					7
**N°4**	18	1	7	Neighbor	None	+	+			+			7
**N°5**	30	3	23	Spouse	None	+	+						14
**N°6**	32	1	32	Spouse	None	+	+	+	+	+			13
**N°7**	20	3	32	Neighbor	None	-	-	-	-	-	+	+	25
**N°8**	22	1	21	Neighbor	None	-	-	-	-	-	+	+	14
**N°9**	38	1	16	Neighbor	None	-	-	-	-	-	+	+	14

## Discussion

There have certainly been more deadly pandemics than that caused by SARS-COV-2 worldwide; however, of all these diseases, COVID-19 is by far the one that has generated more theory and uncertainty both in terms of its origin, its pathophysiology, its clinical symptoms, and its management. Thousands of articles were published worldwide in 4 months; and this paper on a series of pregnant women is one of the first carried out in sub-Saharan Africa. The average age of the patients in our group was lower than those found in the literature [2]. No woman had yet given birth in our series. Logic would suggest, as with other infectious diseases, that maternal physiological changes, lowered immunity, and changes in the cardiopulmonary physiology of pregnancy are responsible for high sensitivity to SARS-COV-2 infection [3,4]. However, no serious form was found in our series. Our results are consistent with those of several studies which have shown that pregnant women have the same symptoms of COVID-19 as the general population [4-6]. Indeed, Chen et al. [5], out of a series of 9 pregnant women, reported no cases of pneumonia or of a critical form, although they all gave birth by cesarean section.

In an another Chinese series, Liu [6] reported fever (77%) and shortness of breath (23%) as the main symptoms. The preterm birth rate was 46% and cesarean section rate was also high in this group of 13 patients (77%). One patient was operated on for ARDS with hepatocellular insufficiency and stillbirth at 34 weeks. As for Zhu et al. [7], after an analysis of a series of 9 patients in Hubei hospitals, found that patients with pneumonia would be more at risk of premature rupture of the membranes, premature deliveries, fetal deaths in utero, delays intrauterine growth and neonatal death. Zhu’s findings are also supported by other authors [8,9,10]. No termination of pregnancy was required in our series. The spontaneous miscarriage in the series cannot be linked to COVID-19; she had flu-like illness in early pregnancy, requiring hospitalization. As the patients are followed, amniotic fluid samples from patients with COVID-19 will be obtained via direct syringe aspiration at the time of delivery. Cord blood and neonatal throat swab samples will be collected immediately after delivery. Additionally, we will collect breastmilk samples from patients with COVID-19 pneumonia after their first lactation. Evidence of mother-to-child transmission will then be evaluated by testing for the presence of SARS-CoV-2 in these clinical samples.

## Conclusion

Pregnant women do not seem to be more at risk of developing severe forms than the general population. This study shows no evidence that pregnant women are more susceptible to infection with coronavirus. However, due to their weaker immune system, caution should be taken towards them

### What is known about this topic

- The influence of Covid-19 in pregnant women is unclear.

### What this study adds

- This study shows that pregnant women are no more at risk than the general population;
- Covid-19 has no detrimental effect on the prognosis of pregnancy.

## Competing interests

The author declares no competing interests.
